# Public Policies and Programs for the Prevention and Control of Breast Cancer in Latin American Women: Scoping Review

**DOI:** 10.2196/32370

**Published:** 2022-07-06

**Authors:** Igor Martín Ramos Herrera, María Guadalupe Lemus Flores, Antonio Reyna Sevilla, Miguel Ernesto González Castañeda, Fernando Adolfo Torres Gutiérrez, René Cristóbal Crocker Sagastume, Juan De Dios Robles Pastrana, José Luis Vázquez Castellanos

**Affiliations:** 1 Department of Public Health University of Guadalajara Guadalajara Mexico; 2 Department of Geography University of Guadalajara Guadalajara Mexico

**Keywords:** breast cancer, scoping review, public policy, prevention programs, systematic review

## Abstract

**Background:**

Breast cancer has positioned itself worldwide as one of the main public health problems, especially in Latin America. In some countries, several programs for the prevention and control of breast cancer in women have been developed and implemented on a permanent basis, but there are no public reports on the policies that originated such programs.

**Objective:**

A scoping review of scientific publications that identify the type, extent, and scope of policies and programs for the prevention and control of breast cancer in Latin American women was performed, and the main results were presented in this paper.

**Methods:**

This scoping review was carried out according to the method by Arksey and O’Malley based on 3 fundamental questions about breast cancer prevention and control policies in Latin America: their type, extent and scope, and reference framework. The search period was from 2000 to 2019, and the search was carried out in the following databases: MEDLINE (PubMed), MEDLINE (EbscoHost), CINAHL (EbscoHost), Academic Search Complete (EbscoHost), ISI Web of Science (Science Citation Index), and Scopus in English, Spanish, and Portuguese, and Scielo, Cochrane, and MEDES-MEDicina in Spanish and Portuguese. Of the 743 studies found, 20 (2.7%) were selected, which were analyzed using descriptive statistics and qualitative content analysis.

**Results:**

The selected studies identified several Latin American countries that have generated policies and programs to prevent and control breast cancer in women, focusing mainly on risk communication, prevention and timely detection, effective access to health services, improvement of the screening process, and evaluation of screening programs. Evaluation criteria and greater participation of civil society in policy design and program execution are still lacking. This could undoubtedly help eliminate existing barriers to effective action.

**Conclusions:**

Although several Latin American countries have generated public policies and action programs for the prevention and control of breast cancer, a pending issue is the evaluation of the results to analyze the effectiveness and impact of their implementation given the magnitude of the public health problem it represents and because women and civil society play an important role in its prevention and control.

**International Registered Report Identifier (IRRID):**

RR2-10.2196/12624

## Introduction

### Background

Breast cancer (BC) has become one of the main public health problems worldwide, especially in Mexico, where, in 2020, a prevalence rate of 225.3 cases per 100,000 women over 20 years was reported [1], whereas the reported mortality was 16 cases per 100,000 women over 20 years. In the same year, 2 out of every 10 cancer-related deaths in women were caused by this particular disease [2]. It was also one of the 5 most outstanding types of fatal cancer among the population aged 30 to 59 years in the period from 2011 to 2016 [2].

In February 2020, BC represented 25% of the cancers diagnosed in women in Latin America (LATAM); hence, of the 462,000 cases that were diagnosed that year, approximately 100,000 resulted in death, of which 56% were women younger than 65 years [3]. This represents 1.8% of the total disability-adjusted life years for women in this region [4].

In this context, the World Health Organization (WHO) predicts that, if the trend of this cancer continues as observed in recent years, by the year 2030, diagnoses will increase by 34% in this region of the planet [5].

By contrast, some studies have identified that breast self-examination and early diagnosis are the best strategies to treat this type of cancer in time [5-8]. Therefore, health education becomes relevant as women can then be trained to actively participate in preventive measures through the adoption of healthy lifestyles, timely identification of gynecological neoplasm warning signs [9], modification of risk factors, and decision to visit a health care facility immediately after identifying any abnormality [10].

In this context, the WHO recommends that countries establish forceful measures (eg, adequate prevention and control policies and programs) to effectively fight BC [5]. In Mexico, as in many other countries, these types of actions are carried out constantly [9,11]. However, there are no comprehensive reports on the diversity, number, type, and scope of programs and public policies that have been implemented or their impact on the population. For this reason, a systematic review, in this case a scoping review, was carried out using the strategy proposed by Arksey and O'Malley [12].

### Objectives

Although there are current scoping reviews about prevention and control issues of chronic-degenerative diseases such as obesity [13], BC [14], or problems generated by little physical activity [15], there is no evidence of a scoping review about policies and programs defining BC prevention and control actions. Therefore, the main objective of this study was to present the results of a scoping review of scientific publications to identify the type, extent, and scope of policies and programs to prevent and control BC in LATAM women to evaluate how these concepts align with existing actions at an international level, identify knowledge gaps, and establish research agendas. The specific objectives of this scoping review were (1) to identify which policies and programs for the prevention and control of BC in LATAM have been analyzed in the last 20 years; (2) to analyze their type, extent, and scope; and (3) to describe the reference frameworks on which these BC policies and prevention and control programs were based.

## Methods

### Design

This scoping review was carried out using the 6-stage methodological framework by Arksey and O'Malley [12], which was later modified by the Joanna Briggs Institute [16]. A research protocol was prepared and registered with the number PRR1-10.2196-12624 at the International Reporting Registry Identifier and later published elsewhere [17]. It was also registered with the Department of Public Health of the University of Guadalajara (CISIGS-021-19) in October 2019. Being a documentary study based on secondary data, it was considered a study without any risk to the population.

### Stage 1: Identification of Research Questions

An iterative search process was conducted to generate one or more questions to guide the investigation. As a result, the Specific Action Program for the Prevention and Control of Cancer in Women 2013-2018 in Mexico was identified [9], whose general objective was used to generate this review’s basic questions considering that they represent the current actions that are being developed in this regard in LATAM (Multimedia Appendix 1).

### Stage 2: Identification of Relevant Studies

#### Overview

The inclusion and exclusion criteria for articles, databases, and search terms were then established. The inclusion criteria were (1) articles on BC prevention and control, public policies, and programs published between January 2000 and December 2019, preferably in Spanish, English, and Portuguese; (2) articles on BC, public policies, and programs applicable to female human participants of any age group; (3) review articles that included systematic reviews, meta-analyses, meta-syntheses, other scoping reviews, and gray literature; and (4) articles published with a focus on the LATAM population. The exclusion criteria were (1) articles on public policies and programs related to any other type of cancer and (2) advertising articles for profit.

#### Databases

The search was carried out in the main electronic databases available internationally and that could be accessed in full text through the Digital Library of the University of Guadalajara [18]. The consulted databases were MEDLINE (PubMed), MEDLINE (EbscoHost), CINAHL (EbscoHost), Academic Search Complete (EbscoHost), ISI Web of Science (Science Citation Index), and Scopus in English, Spanish, and Portuguese, and Scielo, Cochrane, and MEDES-MEDicina in Spanish and Portuguese.

#### Search Terms

For the initial search of BC prevention and control policies and programs, the first filter was “Latin America OR Mexico.” From this initial search, the terms of subsequent consultations included those that were verified in the Medical Subject Headings for the databases in English and in the Health Sciences Descriptors of the Pan American Health Organization for the databases in Spanish and Portuguese. The terms used for the searches were “policies,” “public policies,” “programs,” “strategies,” “laws,” “prevention,” and “control” combined with “Breast Cancer” and “malignant neoplasms” in Spanish, English, and Portuguese.

**Figure 1:**
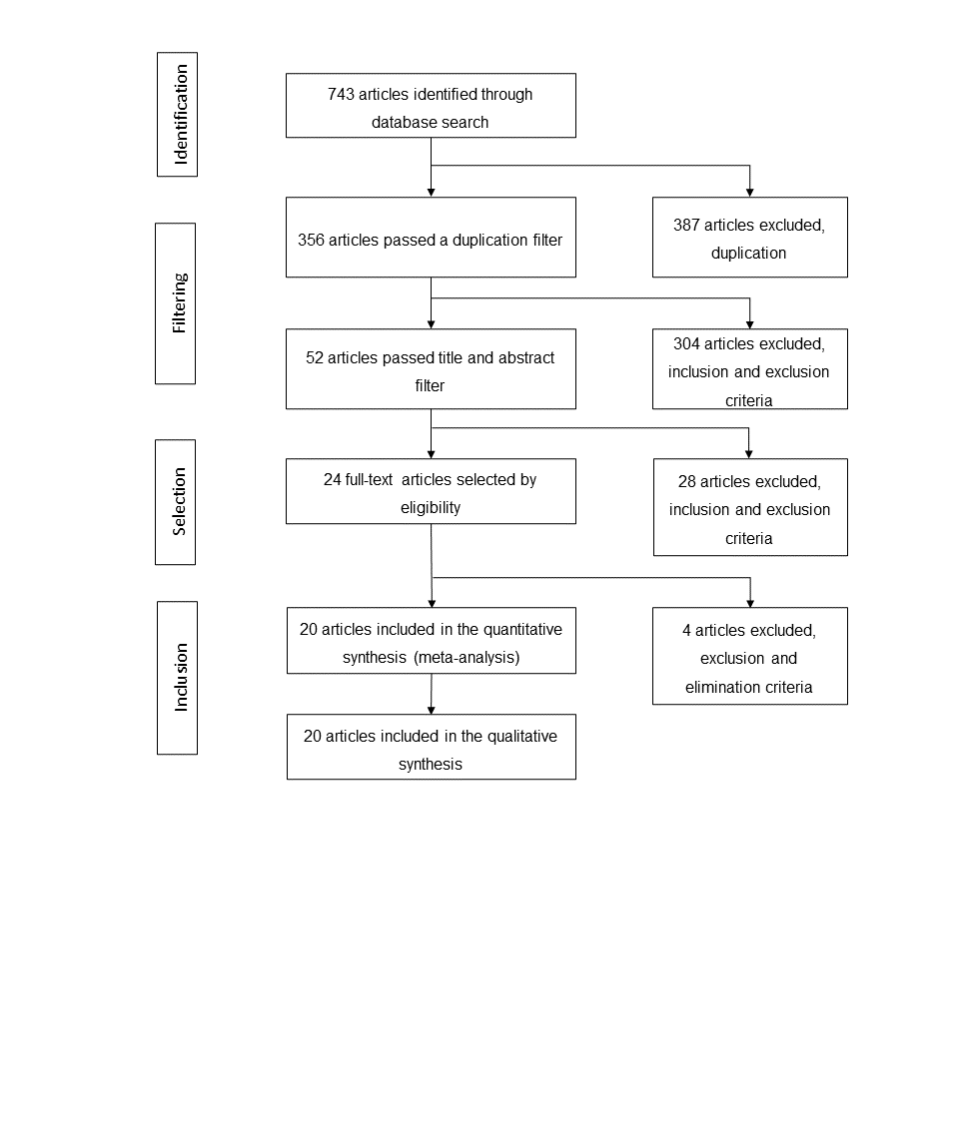
Document identification, screening, and inclusion.

Of interest to this scoping review were those articles that presented information on the policies and programs implemented in LATAM to prevent and control BC. Thus, public policies of the country as well as health education campaigns, promotion of healthy lifestyles, timely detection, and identification of environmental and genetic factors were included in this analysis. Figure 1 shows the steps that were followed and the number of articles that were identified in each step of the process explained previously. As a result of the first step, 743 articles were identified.

### Stage 3: Selection of Studies

In total, 2 researchers (IMRH and MGLF) participated in the search and obtainment of the 743 articles in the previous stage. Titles and abstracts were then reviewed by a group of 3 authors (IMRH, MGLF, and FATG) to identify those that complied with the eligibility criteria; as the end result, of the 743 articles, 20 (2.7%) were selected. Note that 28 (3.8%) articles were excluded at the selection step as they did not comply with the inclusion criteria after full-text reading, and <1% (4/743, 0.5%) were eliminated at the last step after we analyzed them owing to the same criteria. The first researcher reviewed the entire process.

### Stage 4: Data Representation

These 20 articles were then subjected to the data extraction process by the same group of authors (IMRH, MGLF, and FATG plus ARS and MEGC) through specifically designed forms using descriptive statistics and thematic qualitative analysis.

Finally, the articles were organized according to the Specific Action Program for the Prevention and Control of Cancer in Women 2013-2018 [9] and its basic questions (Multimedia Appendix 1). According to this analysis, policies and programs were identified depending on their specific context: geographical, economic, infrastructure, or issues related to BC detection processes.

### Stage 5: Classification of Results, Synthesis, and Report

According to the PRISMA-ScR (Preferred Reporting Items for Systematic Reviews and Meta-Analyses extension for Scoping Reviews) group report guide [19], Table 1 presents the 20 studies that were classified according to the three initially posed questions that, from this point onward, will be treated as analysis categories: (1) BC prevention and control policies, with 5 specific themes; (2) type, extent, and scope of said policies and programs, with 6 specific themes; and (3) reference framework for these policies and programs, with 3 specific themes.

All the studies included in this review (20/20, 100%) analyzed the type, extent, and scope of the addressed policy or program. However, only a few of them explicitly analyzed policies and programs for BC prevention and control (17/20, 85%), mentioned the reference frameworks on which they based their analysis (17/20, 85%), or examined the 3 general categories that guided this review (15/20, 75%).

**Table 1:** Studies included in this review, organized by categories and specific themes for analysis.

Category and themes			Studies
**BC^a^ prevention and control policies and programs**			
	Guaranteeing effective access to quality health services	Agarwal et al [20] Anderson and Cazap [21] Bridges et al [22] Castrezana [23] González et al [24] Knaul et al [25] Niëns et al [26] Nigenda et al [27] Robles and Galanis [28] Torres et al [29] Ulloa et al [30]	
	Improving BC detection and care process	Anderson and Cazap [21] Bridges et al [22] González et al [24] Knaul et al [25] Martínez et al [31] Niëns et al [26] Nigenda et al [27] Robles and Galanis [28] Smith [32] Strasser et al [33]	
	Establishing BC risk communication strategies	Anderson and Cazap [21] Bridges et al [22] Gervas and Pérez [34] González et al [24] Knaul et al [25] Magaña et al [35] Martínez et al [31] Robles and Galanis [28] Smith [32] Tapia et al [36]	
	Focusing on BC prevention and detection actions	Agarwal et al [20] Anderson and Cazap [21] Bridges et al [22] Castrezana [23] Corcoran et al [37] Knaul et al [25] Niëns et al [26] Nigenda et al [27] Robles and Galanis [28]	
	Developing and disseminating performance evaluations of BC screening programs	Anderson and Cazap [21] Gervas and Pérez [34] Knaul et al [25] Martínez et al [31] Robles and Galanis [28] Smith [32]	
**Type, extent, and scope of BC prevention and control policies and programs**			
	BC prevention and control strategies: best practices in line with the sociodemographic characteristics of the populations	Agarwal et al [20] Anderson and Cazap [21] Bridges et al [22] Castrezana [23] Gervas and Pérez [34] González et al [24] Knaul et al [25] Magaña et al [35] Martínez et al [31] Niëns et al [26] Nigenda et al [27] Robles and Galanis [28] Smith [32] Strasser et al [33] Tapia et al [36] Torres et al [29] Valencia et al [38]	
	Reduction of health gaps according to the epidemiological trends of female cancer and the sociodemographic characteristics of the populations	Agarwal et al [20] Anderson and Cazap [21] Bridges et al [22] Strasser et al [33] Torres et al [29] Gervas and Pérez [34] Strasser et al [33]	
	Participation of organized civil society and citizens in processes that improve access to services and actions with political influence (citizen monitoring and supervision)	González et al [24] González et al [39] Knaul et al [25] Nigenda et al [27] Ulloa et al [30]	
	Health service expenses as a responsible investment in relation to the sociodemographic characteristics of the communities	Agarwal et al [20] Anderson and Cazap [21] Bridges et al [22] Knaul et al [25] Smith [32] Strasser et al [33]	
	Systematic monitoring and evaluation to improve BC programs permanently	Agarwal et al [20] Gervas and Pérez [34] González et al [24] González et al [39] Knaul et al [25] Martínez et al [31] Nigenda et al [27] Smith [32] Strasser et al [33]	
	Coordinating the institutions of the national health systems to universalize a BC registry information system and its sources with an ethnic focus and gender perspective to improve epidemiological surveillance	Anderson and Cazap [21] Bridges et al [22] González et al [24] González et al [39] Knaul et al [25] Nigenda et al [27] Robles and Galanis [28] Strasser et al [33]	
**Reference framework for BC prevention and control policies and programs**			
	International BC prevention and control programs	Agarwal et al [29] Anderson and Cazap [21] Bridges et al [22] González et al [39] Knaul et al [25] Martínez et al [31] Niëns et al [26] Nigenda et al [27] Robles and Galanis [28] Smith [32] Strasser et al [33] Ulloa et al [30] Valencia et al [38]	
	National development plans and programs	González et al [24] González et al [39] Knaul et al [25] Martínez et al [31] Niëns et al [26] Nigenda et al [27] Strasser et al [33] Torres et al [29] Ulloa et al [30] Valencia et al [38]	
	Sectorial health plans and programs	Castrezana [23] González et al [24] González et al [39] Knaul et al [25] Martínez et al [31] Niëns et al [26] Nigenda et al [27] Strasser et al [33]	

^a^BC: breast cancer.

## Results

### Quantitative Synthesis

The number of selected studies for each LATAM country was as follows: Argentina (6/20, 30%), Brazil (5/20, 25%), Chile (3/20, 15%), Colombia (4/20, 20%), Costa Rica (2/20, 10%), Cuba (2/20, 10%), Ecuador (1/20, 5%), Mexico (14/20, 70%), Panama (2/20, 10%), Peru (4/20, 20%), Puerto Rico (1/20, 5%), Trinidad and Tobago (1/20, 5%), Uruguay (3/20, 15%), and Venezuela (4/20, 20%). We decided to include a study from Spain (1/20, 5%) [34] as we considered it would be interesting to see how this country, which shares a lot of traditions and culture with LATAM, addressed the BC problem in their female population. Furthermore, some of these studies (14/743, 1.8%) also yielded results from other countries outside LATAM, such as Asia, Australia, Canada, Croatia, Spain and other European countries, India, the Middle East and North Africa, South Africa, and the United States; however, we did not analyze the situation in those countries. We eventually decided to include a study from the United States as it was a systematic review that analyzed the effectiveness of the interventions designed to increase mammography screening in LATAM women residing there.

The number of selected studies by language was 60% (12/20) in English, 40% (8/20) in Spanish, and none in Portuguese. Their general characteristics are shown in Table 2.

The overall objective of each study was analyzed according to their characteristics and design methods. Thus, four different objectives were identified: (1) to generate a guide for the early detection of BC (2/20, 10%), (2) to identify the factors associated with early BC diagnosis (4/20, 20%), (3) to evaluate intervention effectiveness of screening programs or timely detection of BC (9/20, 45%), and (4) to analyze or generate public policies on BC (5/20, 25%).

In addition, of the 20 articles, 4 (20%) reviews and 16 (80%) empirical articles were identified. Of these 16 empirical articles, 11 (69%) were quantitative, and 5 (31%) were qualitative. A total of 40% (8/20) of the studies based their results on international and national health frameworks simultaneously, 30% (6/20) relied exclusively on international health frameworks, and 30% (6/20) relied only on national health frameworks.

Regarding the studies with international health frameworks (14/20, 70%), four sources were specifically identified: (1) the WHO, (2) the International Agency for Research on Cancer, (3) intervention programs from specific countries, and (4) the Breast Health Global Initiative. Only 10% (2/20) of the studies referred to LATAM policies, particularly from the Pan American Health Organization, which are based largely on WHO guidelines.

By contrast, the 65% (13/20) of studies based on a national health framework included the following sources: (1) BC Action Program of the Ministry of Health of Mexico; (2) guidelines of the United States National Cancer Institute; (3) screening programs for the detection of BC in Spain; (4) national programs for BC attention in Mexico (the National Institute of Statistics and Geography, the Ministry of Health, and the National Population Council); (5) Mexico’s Sectorial Health Program from 2007 to 2012; and (6) Official Mexican Regulation SSA-041-2011-2 for the prevention, diagnosis, treatment, control, and epidemiological surveillance of BC (Table 2).

**Table 2:** Characteristics of the selected studies.

Study	Country	Objective	Design and methods	Policy or program addressed	Reference framework
Agarwal et al [20]	Mexico, Croatia, South Africa, and India	Identify possible indicators associated with early diagnosis of BC^a^ in lower-income countries	Quantitative descriptive study; analysis of presented articles about “Breast Cancer Care in Developing Countries” at the International Surgery Week in Montreal, Canada, 2007	International Breast Surgery Program	International: intervention programs
Anderson and Cazap [21]	Latin America	Develop guidelines for the early detection, diagnosis, and treatment of BC in low- and middle-income countries	Review article; variables analyzed: prevention of BC, early detection (self-examination), diagnosis (clinical examination and mammography), and treatment	Breast Health Global Initiative	International: Breast Health Global Initiative; national: National Comprehensive Cancer Network
Bridges et al [22]	Latin America and lower-income countries (Asia, the Middle East, and North Africa)	Identify and compare BC control strategies in Latin America, Asia, the Middle East, and North Africa to develop a common framework to guide the development of national BC control strategies	Qualitative study; 221 semistructured interviews with specialists from 29 different countries on the capacity to train qualified nurses, research infrastructure, and health education	Action program (identified control strategies in the aforementioned countries)	International: WHO^b^
Castrezana [23]	Mexico	Relate the presence of BC in certain geographic spaces with the convergence of environmental and socioeconomic variables	Quantitative analytical study; period: 2000-2012; women younger than 14 years; geospatial analysis of possible risk factors for the development of BC; multivariate regression	No specific program mentioned	National: Breast Cancer Action Program of the Ministry of Health of Mexico
Corcoran et al [37]	United States	Analyze the effectiveness of interventions designed to increase mammography testing of Latin American women residing in the United States	Systematic review; study period: 2009-2011	No specific program mentioned	National: US National Cancer Institute
Gervas and Pérez [34]	Spain	Analyze the effectiveness of health programs that focus on mammography screening	Quantitative, descriptive, observational study; a health action review on BC	No specific program mentioned	National: BC screening programs in Spain
González et al [24]	Argentina, Brazil, Colombia, Mexico, and Venezuela	Analyze the focus of government actions to apply in legislative and operational terms and identify challenges and deficiencies	Literature review; retrospective study; study period: 1990-2008; 90 articles included	BC detection programs in the countries studied	International: IARC^c^
González et al [39]	Argentina, Brazil, Colombia, Mexico, and Venezuela	Analysis of BC care policies and programs in several Latin American countries	Qualitative exploratory study; models used: Hogwood and Gunn; BC prevalence, incidence, and mortality statistics were analyzed; interviews with key actors in the countries indicated	The policies of each country were analyzed, and main national BC care and control programs were included.	International: PAHO^d^ and WHO international reference framework; national: BC national programs of each country
Knaul et al [25]	Mexico	Present world statistics on BC in developing countries, analyze mortality trends in Mexico, and present available data on health care use and access barriers	Descriptive quantitative study based on secondary sources	The Popular Health Insurance Program and the Official Mexican Standard for Cancer Control	International: WHO; national: INEGI^e^, Ministry of Health, and CONAPO^f^
Magaña et al [35]	Mexico	Describe the strategies and actions developed within a training program for the early detection of BC designed for first-level care personnel	Quantitative, experimental, analytical study; evaluation of skills acquired with the training that was implemented from 2008 to 2014	Analyzed the National Medical Education Program for Health Professionals	National: national policies for BC care and control
Martínez et al [31]	Mexico	Analyze BC mortality in Mexico and international recommendations on screening programs; present key aspects of the BC detection and control action program from 2007 to 2012	Qualitative study; health program focused on BC prevention between 2007 and 2012 that covered previous strategies	Breast Cancer Action Program in Mexico from 2007 to 2012	International: WHO and IARC; national: Breast Cancer Action Program in Mexico 2007-2012
Niëns et al [26]	Costa Rica and Mexico	Identify the most cost-effective interventions for BC control in Costa Rica and Mexico from the perspective of medical care	Quantitative study; cost-effectiveness analysis; the average cost-effectiveness ratio of each intervention was calculated	Intervention programs at the IMSS^g^ and the Ministry of Health of Costa Rica	International: WHO
Nigenda et al [27]	Argentina, Brazil, Colombia, Mexico, and Venezuela	Analyze the efforts of 5 Latin American countries in the last 15 years to design and implement BC-related policies	Qualitative study; semistructured interviews with key informants from governmental and nongovernmental organizations; analysis of secondary data from publications in magazines, government reports, and official statistics in each country	Public policies for BC care in the countries included	International: WHO; national: from each country
Robles and Galanis [28]	Latin America, Canada, and the United States	Examine BC mortality in Latin American and Caribbean countries; compare with mortality levels in Canada and the United States; evaluate arguments to develop BC screening programs	Quantitative analytical study; vital statistics records; published data from the cancer registry and information available from the PAHO on disease prevention and control programs, health expenditures, and health service organizations in the region of the Americas	PAHO cancer statistics records of the countries included in the study	International: PAHO, WHO, and IARC
Smith [32]	Latin America, North America, the Middle East, Australia, Asia, and Europe	Analyze BC programs and policies in the countries of the 5 global regions of the WHO to propose programs based on the criteria of the WHO and on each country’s local contexts (type D)^h^	Review study that analyzed national organized screening policies and programs vs opportunistic screening; only low- to middle-income countries were included in the study	Comparative analysis of organized screening policies and programs vs opportunistic screening, mammography, and BC detection programs	International: WHO; national: from each analyzed country
Strasser et al [33]	Latin America	Highlight structural reforms in health care systems, new programs for disenfranchised populations, expansion of national cancer registries, and policy plans and implementation to improve primary prevention of cancer	Quantitative, descriptive, cross-sectional study; health expenditure variables and fragmentation of health systems were analyzed	Health policies that exist in Latin American countries to prevent and control cancer in general were analyzed	International: WHO; national: policies and regulations of each of the countries included
Tapia et al [36]	Mexico	Show teenager perception of BC campaigns	Qualitative study through 13 focus groups	Several BC prevention and national control programs were analyzed	National: Official Mexican Standard for the prevention and control of BC
Torres et al [29]	Mexico	Present the patterns of use of female cancer prevention programs during the 2000 to 2012 period: Papanicolaou test, HPV^i^ test, and mammography	Quantitative, analytical, cross-sectional study; period: 2000-2012; based on national health surveys	BC screening programs using mammography were analyzed	National: Official Mexican Standard for the prevention and control of BC
Ulloa et al [30]	Mexico	Estimate the cost-effectiveness of the BC screening programs and contribute to the decision-making process about the use of these prevention programs	Quantitative, analytical, comparative study through scenario simulation; analysis focused on estimating survival and mortality as well as relating costs to BC diagnosis	The analyzed programs were simulations based on real parameters	International: WHO; methodology for cost-benefit analysis
Valencia et al [38]	Mexico	Estimate the cost-effectiveness ratio of BC prevention programs	Quantitative, analytical, cross-sectional study based on the Markov model with 4 processes: the natural evolution of BC, BC detection through mammography screening, BC treatment, and dynamics of mortality from other causes	BC prevention and control policies in Mexico were analyzed	International: WHO; national: BC prevention and control programs in Mexico

^a^BC: breast cancer.

^b^WHO: World Health Organization.

^c^IARC: International Agency for Research on Cancer.

^d^PAHO: Pan American Health Organization.

^e^INEGI: National Institute of Statistics and Geography.

^f^CONAPO: National Population Council.

^g^IMSS: Mexican Institute of Social Security.

^h^The type of objective indicated in parentheses in the description of each objective corresponds to the classification made by the authors, which is presented in the *Results* section.

^i^HPV: human papillomavirus.

### Qualitative Synthesis

#### Overview

For a better understanding, the selected studies were analyzed according to the 3 previously defined general categories (Table 3) based on the Specific Action Program for the Prevention and Control of Cancer in Women 2013-2018 [9].

**Table 3:** Breast cancer care policies and programs reported in the selected studies.

Study	Type	Name of the analyzed public policy and action program	Scope: level of care	Framework
Agarwal et al [20]	Program	The National Breast Cancer Screening Program	First and third level of care	International
Anderson and Cazap [21]	Program	BHGI^a^; NCCN^b^	First, second, and third level of care	International: BHGI; national: NCCN
Bridges et al [22]	Public policy	Breast cancer control strategies in the studied countries; WHO^c^	First, second, and third level of care	International: WHO
Castrezana [23]	Program	Breast Cancer Action Program of the Ministry of Health of Mexico	First level of care	National: Ministry of Health
Corcoran et al [37]	Program	Breast Cancer Action Program of the US Department of Health and Human Services	First level of care	National: US Department of Health and Human Services
Gervas and Pérez [34]	Program	Secondary prevention program; National Cancer Institute of the United States	Second level of care	National: National Cancer Institute
González et al [24]	Public policy and program	Argentina (Early Detection of Genito-Breast Cancer Program and Oncological Diseases Program); Bolivia (Noncommunicable Disease Prevention and Control Management Plan 2005-2009); Brazil (National Cervical and Breast Cancer Control Program “Viva Mulher”); Chile (National Breast Cancer Program); Colombia (National Breast Cancer Program); Mexico (Breast Cancer Action Program 2007-2012); Panama (Comprehensive Women’s Health Program); Peru (National Plan to Strengthen Cancer Prevention and Control in Peru); Uruguay (Breast Cancer Early Detection Program); Venezuela (National Breast Cancer Program)	First, second, and third level of care	National: several Latin American countries
González et al [39]	Public policy and program	Argentina (National Cancer Control Program, Breast Cancer Secondary Prevention Subprogram, Compulsory Medical Program, and Program for the Early Detection of Genito-Breast Cancer); Brazil (National Oncology Policy 2439, Comprehensive Women Health Care National Policy, and “Viva Mulher” Program 1998); Colombia (7 Procedures and Interventions Manual and Basic Plan of Care with technical standard for breast cancer detection); Mexico (Specific Action Program); Venezuela (Breast Cancer Control Subprogram)	First, second, and third level of care	International: IARC^d^
Knaul et al [25]	Program	“Oportunidades” program	First level of care	International: WHO; national: INEGI^e^, Ministry of Health, and CONAPO^f^
Magaña et al [35]	Public policy	National breast cancer care and control policies	First level of care	National
Martínez et al [31]	Program	Breast Cancer Action Program; Mexico’s Sectorial Health Program from 2007 to 2012	First, second, and third level of care	International: WHO and IARC; national: Mexico’s Sectorial Health Program
Niëns et al [26]	Public policy	Policies from international organizations	First and third level of care	International: WHO
Nigenda et al [27]	Public policy	Policies from international organizations	First level of care	International: WHO; national: several countries
Robles and Galanis [28]	Public policy	Policies from international organizations	First level of care	International: WHO; national: several countries
Smith [32]	Public policy	Health Insurance Plan of Greater New York; Swedish Board of Health and Welfare; the Breast Health Global Initiative; Mexican Foundation for Education in Prevention and Opportune Detection of Breast Cancer	First level of care	International: WHO; national: several countries
Strasser et al [33]	Public policy	General policies of Latin American countries	First and second level of care	International: WHO; national: several countries
Tapia et al [36]	Program	Alliance with companies; Prevention is in our hands: sit down and explore yourself; Mom, we go together; Save them all and take care! Jalisco wants you alive; Please Touch; mobile units	First level of care	National: Official Mexican Standard for the prevention and control of breast cancer
Torres et al [29]	Program	Breast cancer screening program with mammography, Papanicolaou smear, and HPV^g^ test	First and third level of care	National: Official Mexican Standard for the prevention and control of breast cancer
Ulloa et al [30]	Public policy	Methodology for cost-benefit analysis of international organizations	First and second level of care	International: WHO
Valencia et al [38]	Public policy and program	Policies of international organizations; breast cancer prevention and control programs in Mexico	First level of care	International: WHO; national: Ministry of Health

^a^BHGI: Breast Health Global Initiative.

^b^NCCN: National Comprehensive Cancer Network.

^c^WHO: World Health Organization.

^d^IARC: International Agency for Research on Cancer.

^e^INEGI: National Institute of Statistics and Geography.

^f^CONAPO: National Population Council.

^g^HPV: human papillomavirus.

#### Category 1: BC Prevention and Control Policies and Programs

##### Overview

The reviewed studies identified that many LATAM countries have developed several BC prevention and control policies and programs. All of them have focused on educational actions and implementing screening tests with different strategies depending on each country’s situation. However, we could identify that not all studies presented the results of their implementation, and those that did showed great differences in scope and impact (Table 3). With the applied content analysis, 5 specific themes could be identified (Table 1).

##### Establishing BC Risk Communication Strategies

The reviews identified the lack of knowledge that the general population has regarding early BC prevention and diagnosis (eg, key symptoms, genetic inheritance, screening methods, time intervals to perform surveillance and control examinations, risk factors, and late clinical stages [22,24,32,34,36]).

Therefore, some studies proposed educational intervention programs for target populations to disseminate useful information and educate the population with the highest BC incidence [22,24,25,34]. In this sense, it is essential to design training programs for health professionals as they are the ones who can educate patients and communities by explaining, for example, what BC is about or by encouraging periodic screening [24,32,35,36].

##### Focusing on BC Prevention and Detection Actions

On this topic, the selected studies proposed prevention strategies for the community through education on risk factors, exploration methods, and identification of the disease’s early signs [20,21,23,26,27], thus highlighting the importance of offering mammography tests at the first level of care (ie, community health centers). Moreover, the need to create official policies and programs to adequately allocate the economic resources for these actions was acknowledged [22,25-28].

##### Guaranteeing Effective Access to Quality Health Services

A main and frequently mentioned element was the deficient access to quality health services for BC care in women, especially for those who lack economic resources, reside in rural areas, or have a lower academic level [20]. Another identified element was the lack of government funding that would allow, among other things, for the reduction of the BC mortality rate attributable to deficient health service access [27]. In addition, health care centers in rural communities require technology to follow up on probable or confirmed patients as well as computer systems designed for this purpose, which currently do not exist [21,22,24,30].

##### Improving BC Detection and Care Process

The selected studies referred to the benefits of early BC diagnosis and timely treatment to reduce mortality and increase life quality [25-27,31]. Therefore, they focused on improving detection methods and increasing them in accordance with national and international recommendations (eg, through mammography screening or clinical examination [21,27,28,37]). In addition, the importance of training health professionals to perform BC detection in time was emphasized [32,33].

##### Developing and Disseminating Performance Evaluations of BC Detection Programs

The studies emphasized the importance of implementing health programs for BC prevention and control. However, only a few presented an impact evaluation even though several were contradictory; that is, the studies by Anderson and Cazap [21], Knaul et al [25], and Martínez et al [31] reported successful programs, whereas others presented only limited results. Therefore, it is necessary to establish evaluation indicators in national programs to know their effectiveness and impact [28,32].

#### Category 2: Type, Extent, and Scope of BC Prevention and Control Policies and Programs

In total, 6 specific themes were identified in relation to how policies were defined and their content (Table 3), as explained in the following sections.

##### BC Prevention and Control Strategies: Best Practices in Line With the Sociodemographic Characteristics of the Populations

On this topic, several studies highlighted the relevance of generating health action plans and programs focused on providing quality information to the population about risk factors, screening measures, control and prevention, and treatment but adjusted to age groups and the socioeconomic conditions of women in such a way that the programs and plans comply with specific needs and conform to the best available evidence [22,24,25,31,35,36]. By contrast, they also included the issue of innovation in programs that train new generations of health professionals in the correct identification of BC early stages; the programs must include both a health care component (technical and clinical) and an administrative component to be successful [22-29,31-36,38].

##### Reduction of Health Gaps According to the Epidemiological Trends of Female Cancer and the Sociodemographic Characteristics of the Populations

The studies referred to the great differences that may exist in several countries regarding women’s access to health services depending on their socioeconomic level, whether it is high or low; mortality rates are generally higher in the latter. For example, it is possible that, because of lacking financial resources to obtain an early diagnosis and timely treatment, the region where these women live may not have sufficient technology to provide that, thus forcing them to invest in those services themselves, which could lead patients at risk to decide not to make such investment [20-22,29,33]. Another reason why adequate BC control is not carried out is the lack of mammography equipment in marginalized locations, which compels patients to travel to distant cities and increases the delay in their diagnosis [33,34].

##### Participation of Organized Civil Society and Citizens in Processes That Improve Access to Services and Actions With Political Influence (Citizen Monitoring and Supervision)

There was consensus regarding the operation of strategies that involve the general population, including remote or marginalized areas, in the design and implementation of BC promotion and prevention programs [27] and the identification of communication deficiencies, provided information, and health services to meet the needs of different age groups as well as adequately use the economic resources assigned by the government to acquire equipment that could really increase the impact of screening [24,25,27,30,39]. An example of this is Brazil, where women of different ethnicities are frequently included in the creation of health programs that focus on improving BC detection and passing on information in their region [27]. By contrast, some civil society initiatives draft policies and define arrangements with organizations in some LATAM countries such as Brazil, Colombia, and Mexico where interaction between legislators, authorities, groups of interest, and the community exists [24,27,39]. However, despite this interaction, more spaces for participation are needed. In these same countries, inclusive participation in the decision-making of governmental and nongovernmental institutions has been proven, whereas, in Venezuela and Argentina, the greatest influence comes from the government.

##### Health Service Expenses as a Responsible Investment in Relation to the Sociodemographic Characteristics of the Communities

The studies reported high treatment and control costs for patients with advanced BC in public institutions [22] in contrast to the expenses of private institutions that offer screening and control programs that can detect the disease at early stages, which in turn reduces mortality and costs. Another important element is health system saturation in LATAM countries as well as budgetary restrictions, which often lead to treatment delays and favor the progression of the disease [33]. Therefore, the need to invest in programs and action plans according to the context of each country (burden of disease, sociodemographic and epidemiological characteristics, and available resources) was highlighted, and that includes an articulated social response. In this way, the supply of health services could expand, public spending could be reduced, and life quality in communities could improve. Compared with other regions in the world, LATAM in general is not well-equipped to cope with the alarming increase in cancer incidence and the disproportionately high mortality rates [20,33].

##### Systematic Monitoring and Evaluation to Improve Programs Permanently

The need to establish evaluation standards and parameters for BC screening programs in the community, including physical examination by highly trained health professionals in mammography screenings, is evident as several authors did not approve of screenings performed with only one of these methods because they considered them inefficient [24,34], which implies that the programs must be standardized [27,32,33,39]. As a result of this, it would be possible to measure the effectiveness, costs, and impact of such standards and parameters [31]. The most common mistake in evaluating screening program effectiveness is not recognizing that the general population may be different from the population that is susceptible to screening [20,25].

##### Coordinating the Institutions of the National Health Systems to Universalize a BC Registry Information System and Its Sources With an Ethnic Focus and Gender Perspective to Improve Epidemiological Surveillance

Through this review, deficiencies in case reporting and registry systems were identified as well as the lack of histopathological reports identifying the BC clinical stage in which women first attended health services, which leads to incomplete clinical records that result in difficult clinical decision-making processes by the health sector to implement a correct strategy for these populations [22,33]. The main factors that generate this situation are (1) lack of reliable data on prevalence and incidence at the national level in most LATAM countries because of the lack of national population-based registries [24,25,39], (2) establishment of quality measures to provide institutions with the necessary equipment to perform diagnoses and reports properly, and (3) clinical underregistration because of the difficulty of achieving early detection [28]. Therefore, achieving reliable statistics, comparative evaluations, high-quality national registries, and epidemiological and ethnic statistics as well as improving the capacity of information systems (eg, the use of technology and adequate data management) is essential [21,27].

#### Category 3: Reference Framework for BC Prevention and Control Policies and Programs

In this last category, three themes are described detailing the level at which the studies addressed their analysis: (1) international BC prevention and control programs, (2) national development plans, and (3) sectorial health programs. We will review them in detail.

##### International BC Prevention and Control Programs

This topic brings together most of the health policies, programs, and actions for BC screening and control made by international organizations such as the International Agency for Research on Cancer, which indicates the deficiencies of lower-income countries as opposed to higher-income countries that have better BC control [20,39]. By contrast, it is recognized that most LATAM countries have standards, laws, decrees, and regulations that establish actions and interventions for the early detection, diagnosis, treatment, and follow-up of the population with the disease [20-22,32,33].

In terms of investment, to guarantee screening and treatment coverage for the population and motivate women to participate in screening tests [32], the analysis showed that the greater the access to health institutions, the lower the BC mortality. Among the institutions that design international recommendations, we found the Breast Health Global Initiative [21], which strives to develop guidelines based on economically feasible and culturally appropriate evidence that can be used by countries with limited resources. There is also the Commission on Macroeconomics and Health of the WHO [22,25-28,30,31,33,38], which, based on the gross domestic product, established thresholds where a cost-effective intervention can be considered.

##### National Development Plans and Programs

Health programs offered in most LATAM countries are designed through government initiatives depending on the structure of their health systems. In countries with fragmented systems such as Mexico, health institutions offer these services [11]. For example, the Institute for Social Security and Services for State Workers offers screening services to populations that belong to the government’s workforce, and the Popular Security Program protects populations that have neither of the aforementioned services [24,38,39].

In addition, these studies mentioned health regulations, which include the standards that must be followed to make a proper diagnosis, the average age that patients must be to attend health services, and the treatment that must be offered for each case. In terms of gross domestic product per capita, in 2007, Mexico’s expenditure ranged between 0.7 and 1.6 percentage points, which falls within the range of a cost-effective expenditure per life year according to WHO recommendations [25-27].

Some of the implemented programs were also based on national policies, such as the National Program for Sexual Health and Responsible Procreation in Brazil [27]; the Ministerial Resolution 0903 of December 20, 2004, and the 2005-2009 Prevention and Control Plan for Noncommunicable Diseases in Bolivia [24]; Law 19,966, General Regime of Health Guarantees and Supreme Decree 44 of January 2007 in Chile [24]; Resolution 00412 of 2000 and the Technical Standard for the Early Detection of Breast Cancer in Colombia [27]; a set of Benefits of the National Health System based on the 2006 resolution of the National Health Council Directory in Ecuador [24]; the Official Mexican Standard (NOM-041-SSA2-2002) for BC prevention, diagnosis, treatment, control, and epidemiological surveillance in Mexico [29]; the Comprehensive Care Standard for Women, BC Detection Component established in Panama [24]; the Headquarters Resolution 121 in 2008 and the technical-oncological standard for the prevention, detection, and early diagnosis of BC at the national level released in Peru [24]; the Executive Power Decree 202/005 that defines the National Cancer Control Program released in Uruguay [27]; and the Program for the Early Detection of Genito-Breast Cancer and Sexually Transmitted Diseases in Argentina [24]. By contrast, the studies also commented on the deficiencies of health institutions and action programs, which make BC difficult to diagnose in the early stages, and the lack of equipment and human resources to follow up and control this pathology in public institutions [30,31,33].

##### Sectorial Health Plans and Programs

The selected studies mentioned that health programs with intersectoral involvement chose to divide the action programs, which previously addressed various gynecological pathologies, into specific programs that exclusively treat BC [24,39]. Some mentioned that the process of legitimizing politics through normative action is the one that has advanced the most in the region [31].

Consequently, there is a wide range of extensive and inclusive normative and regulatory frameworks (regarding population and actions to control the disease) as well as clinical management guidelines and protocols agreed upon by health authorities, academic associations, scientists, and civil organizations in all the countries, Mexico in particular [23,27]. Therefore, nongovernmental organizations play an important role in the development and implementation of an integrated response. Although there are several organizations that specialize in BC and work to provide information and raise awareness, this condition seems to be much less integrated in nongovernmental organization programs that provide other types of services to women at risk [25,26,33].

## Discussion

### Principal Findings

We identified that the studies included in this review analyzed different perspectives on the design and implementation of BC programs and public policies, as reported in the quantitative section of the analysis. The studies focused mainly on risk communication, prevention and timely detection, effective access to health services, improvement of the detection process, and evaluation of screening programs, the latter being the topic that received the least attention. It is useful to generate policies and programs aimed at addressing women’s BC problem; however, whether they function properly or have the expected impact in each country and on the sociodemographic characteristics of women must also be evaluated.

BC is an issue of interest to health authorities and governments worldwide. The approach and resources set aside for its attention are similar in different regions, such as Asia, the Middle East and North Africa, Australia, and North America, as reported by Bridges et al [22]. According to these authors, there are 4 themes that comprise the foundation for national BC control strategies in these regions: building capacity, developing evidence, removing barriers, and promoting advocacy. They also found that the discussion of these matters and their dimensions was similar across the regions. However, in Australia and Canada, managing advocacy was discussed more frequently, and organized advocacy was discussed less frequently. On the same line, the experience of local practitioners in different regions is made clear in the comment by Smith [32]: “There is consensus that programs should be designed based on disease burden and available resources, but that even in low resource countries there are opportunities to reduce breast deaths through earlier diagnosis and effective treatment. Screening programs are most effective when they are organized, and program planners should consider WHO criteria and local input data as a basis for tailoring screening programs to the needs of their population.” These 2 studies emphasize that the design of new policies and programs should be grounded on the experience of local practitioners, policy makers, and advocacy leaders throughout the regions of the world.

In LATAM countries, the existing barriers that were reported in several studies (9/20, 45%) were the scarcity of funds and the lack of well-prepared human resources to carry out the program’s follow-up and to control the disease in public health institutions, as well as the barriers related to the characteristics of the women to whom they are directed and the social determinants that affect them (ie, educational level, marginalized areas, or areas with poor health care accessibility). Furthermore, most LATAM countries still have a fragmented health care system, which involves several separate health coverage schemes and providers. On the one hand, this refers to adequately financed social security systems that insure formally employed people, including cancer treatment coverage. By contrast, there is financially limited health security with poorly managed coverage or without full coverage for the entire population [30]. Finally, there is a large amount of private health services that the population in general can access, even those with low economic resources.

We also found that the situation is not the same for all countries in the region as there are big economic, social, and geographical differences that hinder the implementation of effective programs even though most of the countries have developed BC prevention and control policies in the last 20 years. For instance, according to the World Bank, some of the countries are classified as high-income economies, such as Chile, Uruguay, and, in the case of this review, the United States and Spain. Others belong to upper–middle-income economies, such as Argentina, Brazil, Guatemala, Mexico, and the other 6 countries, and the rest of the region’s countries are lower–middle-income economies, such as Bolivia, El Salvador, Honduras, and Nicaragua. No country in the LATAM region is classified as a low-income country [40]. By contrast, some countries have capitalist governments, whereas others have socialist or military governments. Finally, there are large demographic differences as some countries have more than 100 million inhabitants (Brazil and Mexico), whereas other countries have less than 5 million inhabitants (Bahamas, Panama, and Puerto Rico), and their geographical extensions are extremely different as well [40]. All of these elements affect the implementation of policies and programs, and they have a direct impact on BC prevalence and the efficiency with which the problem is addressed. For example, Strasser et al [33] report that countries such as Costa Rica, Chile, Colombia, and Brazil have implemented universal health care systems with high cancer coverage to avoid catastrophic expenses, as well as more screening and detection programs. These countries have reached health coverages of less than 90%, whereas Mexico only covers 45% of its population.

Other indicators, such as palliative care services that reflect the efforts made by some LATAM countries, show that Argentina has recently integrated palliative care courses into training programs; however, Bolivia, El Salvador, Honduras, and Nicaragua still have no specific training in that area. Until recently (2014), there were no action plans, strategies, or policies for cancer care and control in Peru, Ecuador, Belize, Surinam, El Salvador, Panama, and the Dominican Republic; however, they are currently developing national strategies (Peru) or specific programs (El Salvador and Ecuador).

We can point out that the main challenge for LATAM countries is to develop comprehensive and adequately structured BC programs with appropriate resources even though some countries already include them in broader care programs for women [25,27,31,39], such as the initiative proposed by the WHO called the Global Breast Cancer Initiative [41], which aims to reduce BC burden by 25% per year to achieve the goal of saving 2.5 million lives by 2040 and offers guidance to the governments on how to adapt their health care systems and empower preventive measures. Other countries report higher rates of BC detection in late stages [25] owing to the big differences that still exist between different types of insurance coverage for the population, which results in the persistence of fragmented health systems in countries such as Peru, Colombia, and Mexico [33]. Only Brazil, Cuba, and Costa Rica claim to have universal health systems.

To define policies and programs, it is important to consider the predictors of a delayed BC diagnosis. The scoping review by Webber et al [8] reports that the main predictors are those provided by the patient and the diagnosis intervals. This situation is related to the efforts that should be made by health authorities to improve awareness of BC symptoms and encourage disclosure, which could improve timely BC diagnosis as well as provide access to health services for vulnerable groups, all of which should be addressed in programs and policies. These results are similar to what we found in our study. Although studies from countries with high sociodemographic indexes in the American continent, such as the United States or Canada [4], were excluded from this study, those governments and populations are likely to face different pressures regarding access to BC care.

Finally, some of the following are limiting problems that we found in this review: there is no BC follow-up for all women, particularly those belonging to low socioeconomic strata and without health insurance [30]; evidence-based standards must be developed according to the economic and cultural reality of each country [38]; screening programs must be applied to reach an incidence reduction of up to 35% in women who undergo mammography screenings regularly [31]; and, finally, there is limited evidence showing that mammography screening is cost-effective for patients with different income levels [22,25].

### Conclusions

LATAM countries have made important efforts to face BC burden and mortality in their populations. However, there is an imperative need to continue those efforts and even develop more programs but based on coherent and comprehensive public policies. In addition, programs and screening efforts should be evaluated after their implementation, and the results should be made publicly available for those who participated and for all women in general in the hope that numbers decrease according to the sustainable development goals [1].

In this context, this review’s results will be used to define the grounds for a research project whose purpose is to propose a public policy that supports the design of educational intervention strategies for the open population in Mexico to address the public health problems that were identified in this study. Similarly, this review identified some of the gaps that still exist in public health policies and contribute to the underdevelopment of comprehensive, properly structured, and financed BC programs [24]. Finally, these results, in the end, will be presented to national and international health legislators as a guide to promote the necessary public policies for LATAM countries.

## Appendix

Multimedia Appendix 1 Research questions and operational definitions. Document article identification, screening, and inclusion.

## Abbreviations

BC: breast cancer
LATAM: Latin America
PRISMA-ScR: Preferred Reporting Items for Systematic Reviews and Meta-Analyses extension for Scoping Reviews
WHO: World Health Organization
