# Supplementary material for: Public Policies and Programs for the Prevention and Control of Breast Cancer in Latin American Women: Scoping Review
Source: JMIR Cancer. 2022 Jul 6;8(3):e32370. doi: 10.2196/32370 (PMC9301550; doi:10.2196/32370)
Supplement: Multimedia Appendix 1 [file cancer_v8i3e32370_app1.docx]

Multimedia Appendix

Multimedia Appendix 1. Research questions and operational definitions.

| **Research questions**  **(Categories)** | **Operational definitions**  **(Themes)** |
| --- | --- |
| What policies for the prevention and control of BC in Mexico have been analyzed in the national and international scientific literature? | 1. Establishing BC risk communication strategies 2. Focusing on BC prevention and detection actions 3. Guaranteeing effective access to quality health services 4. Improving BC detection and care process 5. Developing and disseminating performance evaluations of BC screening programs. |
| What is the type, extent and scope of those policies/programs according to the reports? | 1. BC prevention and control strategies: best practices in line with the sociodemographic characteristics of the populations 2. Participation of organized civil society and citizens in processes that improve access to services and actions with political influence (citizen monitoring and supervision). 3. Health services expenses, as a responsible investment, in relation to the sociodemographic characteristics of the communities. 4. Systematic monitoring and evaluation to permanently improve BC programs. 5. Coordinating the institutions of the National Health Systems to universalize a BC registry information system and its sources, with an ethnic focus and gender perspective, to improve epidemiological surveillance. |
| What is the reference framework for BC prevention and control policies/programs at national and international levels? | 1. International BC prevention and control programs. 2. National development plans and programs. 3. Sectorial health plans and programs. |
